# Overexpression of the Transcriptional Regulator *WOR1* Increases Susceptibility to Bile Salts and Adhesion to the Mouse Gut Mucosa in *Candida albicans*

**DOI:** 10.3389/fcimb.2017.00389

**Published:** 2017-09-12

**Authors:** Daniel Prieto, Elvira Román, Rebeca Alonso-Monge, Jesús Pla

**Affiliations:** Departamento de Microbiología II, Facultad de Farmacia, Universidad Complutense de Madrid Madrid, Spain

**Keywords:** *Candida albicans*, gut colonization, GUT, Wor1, adhesion

## Abstract

The transcriptional regulator Wor1 has been shown to induce the GUT transition, an environmentally triggered process that increases the fitness of *Candida albicans* in the mouse gastrointestinal tract. We have developed strains where the expression of this gene is driven from the strong and tightly regulated tetracycline promoter. These cells retain the main characteristics reported for GUT cells albeit they show defects in the initial stages of colonization. They also show a differential colonization along the gastrointestinal tract compared to isogenic strains, which is probably caused by their susceptibility to bile salts. We also show that *WOR1* overexpressing cells have an altered metabolic activity, as revealed by a different susceptibility to inhibitors of respiration, and an enhanced adhesion to the mouse mucosa. We propose that this may contribute to their long-term favored ability to colonize the gastrointestinal tract.

## Introduction

The fungus *Candida albicans* is a frequent colonizer of the human gastrointestinal and female vaginal tract. It is estimated that more than 50% of human individuals without an underlying pathology are colonized with this fungus and this value may be higher as colonization is highly dependent on the physiological status of the patient. Broad-spectrum antibacterial antibiotics, diabetes, and immunological disorders (among others) favor overgrowth of *C. albicans* in certain niches. Alteration of the host defenses facilitates the access of the microbe to other non-canonical body locations causing severe diseases called candidiasis. These conditions are, frequently, life threatening and may result in mortalities as high as ≈50% (Gudlaugsson et al., 2003). While the identification of virulence factors responsible for infection has been a major goal in the recent years (Navarro-García et al., 2001; Mayer et al., 2013) the identification of those factors that promote colonization is also central to fungal research. Therapies directed against the associated genes and proteins or the processes involved may lead to the eradication of *C. albicans* from the gut or to restrict its presence as a “domesticated” commensal, both of potential usefulness in the prevention of candidiasis.

The implementation of animal colonization models in recent years (see Koh, 2013 for a review) has enabled the identification of processes involved in the adaptation of *C. albicans* to the commensal state (Neville et al., 2015; Noble et al., 2016; Prieto et al., 2016). Iron and glucose metabolism, signal transduction, and morphogenetic transitions (Pierce and Kumamoto, 2012; Noble, 2013; Perez et al., 2013; Prieto et al., 2014; Vautier et al., 2015; Ramírez-Zavala et al., 2017) have all been revealed critical for the ability of *C. albicans* to colonize the mouse gut in antibiotic treated mice. Pioneer studies revealed that mutants altered in the Efh1 transcription factor showed increased colonization over wild type (wt) cells and that overexpression of *EFH1* led to reduced colonization (White et al., 2007). Further studies revealed that the Efg1 morphogenetic regulator (Stoldt et al., 1997) also played a role in commensalism as *efg1* mutants outcompeted wt cells at early time points after colonization, a phenomena that was not maintained at later time points (Pierce and Kumamoto, 2012). Efg1 is also an important regulator of the white opaque (**wo**) transition (Sonneborn et al., 1999), an environmentally regulated genetic program that prepares cells for mating (Soll, 2014). The **wo** switching is repressed by the **a**1-α2 repressor, and therefore, only occurs in **a** or α cells. It is triggered by environmental signals (Morschhauser, 2010) and it is favored by low (21°C) temperatures. The *WOR1* gene (master regulator of the wo transition) was identified as **a**1–α2 repressed gene in the white phase whose deletion blocked opaque formation (Zordan et al., 2006). Overexpression of *WOR1*, on the contrary, provokes an en-masse conversion of the white population to opaque cells (Huang et al., 2006). Recently, an unusual switch specific of the adaptation to the commensal lifestyle has been described. The GUT (Gastrointestinally indUced Transition) transition was observed upon the passage of *C. albicans* cells overexpressing *WOR1* along the mouse gastrointestinal tract (Pande et al., 2013). Deletion of *WOR1* caused a reduction in fitness while overproduction of this protein from the strong *TDH3* promoter (*WOR1*^OE^) increased it. GUT cells could be differentiated from “standard” opaque cells by its surface ultrastructural details (absence of pimples present in opaque cells), transcriptomal analysis and *in vivo* fitness.

The susceptibility of GUT cells to stresses associated with gut colonization including bile salts, antimicrobial peptides or reactive oxygen species (ROS) is still not known. Therefore, it would be interesting to determine the mechanisms promoted by *WOR1* in a complex niche where multiple signals are constantly being generated by a combination of host microbiota and nutrients (among others).

In this work, we have undertaken an analysis of GUT-like cells both *in vitro* and during *in vivo* colonization. We show that soon after oral inoculation, GUT-like cells suffer a decrease in cell viability, which is probably caused by their enhanced bile salt sensitivity. We also reveal metabolic and adhesion alterations associated with GUT-like cells that modulate their ability to colonize the mouse tract, features that may be critical to their role in commensalism.

## Results

### High *WOR1* expression develops a gut-like phenotype *in vitro*

To generate GUT-like *C. albicans* cells we overexpressed the gene *WOR1* in **a/**α *MTL* background, as reported elsewhere (Pande et al., 2013). For this purpose, we chose in this study the doxycycline regulated promoter (Park and Morschhauser, 2005) to express *WOR1*, as it represents a strong promoter widely used in *C. albicans* research and, most importantly, because of its ability to regulate gene expression *in vivo*. Therefore, a genetic construction carrying a myc-epitope tagged version of *WOR1* regulated under the TET-OFF system was integrated at the *ADH1 locus* in *C. albicans*. The strain obtained, CAI4-*WOR1*^*OE*^, was shown to produce Wor1-myc as a protein of ≈90 kDa whose expression was doxycycline-dependent and similar to the 84.44 kDa predicted molecular weight of the construct (Supplementary Figure 1A). In addition, CAI4-*WOR1*^*OE*^ retained *MTL* heterozygosity (Table 1 and Supplementary Figure 1B) excluding the possibility that the transformation altered the mating type (Selmecki et al., 2010). We observed that CAI4-*WOR1*^*OE*^ cells and colonies *in vitro* appeared as opaque. The colonies stained with phloxine B, as opaque cells, (Table 1 and Supplementary Figure 1C) and pimples were absent at the cell surface. This constrasts with opaque cells where these structures are typically found (Table 1 and Supplementary Figure 1D). Phloxine B positive phenotype was stable over time and independent of the temperature (37 or 21°C). The addition of doxycycline reversed en-masse the population to phloxine B negative cells, even at 21°C. This effect was reversed upon removal of doxycycline (Supplementary Figure 1E).

**Table 1:** Main characteristics of opaque and GUT cells.

	**Opaque (WO-1)**	**GUT (as determined in Pande et al., 2013)**	**GUT-like (this work)**	**Opaque (as determined in Xie et al., 2013)**
Phloxine B staining	+	ND	+	+
*MTL* status	Homozygosis	Heterozygosis	Heterozygosis	Heterozygosis
Stability at 37°C	–	+	+	–
*WOR1* overexpression	“Physiological”	Strong ectopic (*TDH3*^PR^)	Strong ectopic (*TET*^PR^)	Variable levels
Pimples	+	–	–	+
Development	*in vitro*	*in vivo*	*in vitro*	*in vitro*

**Figure 1:**
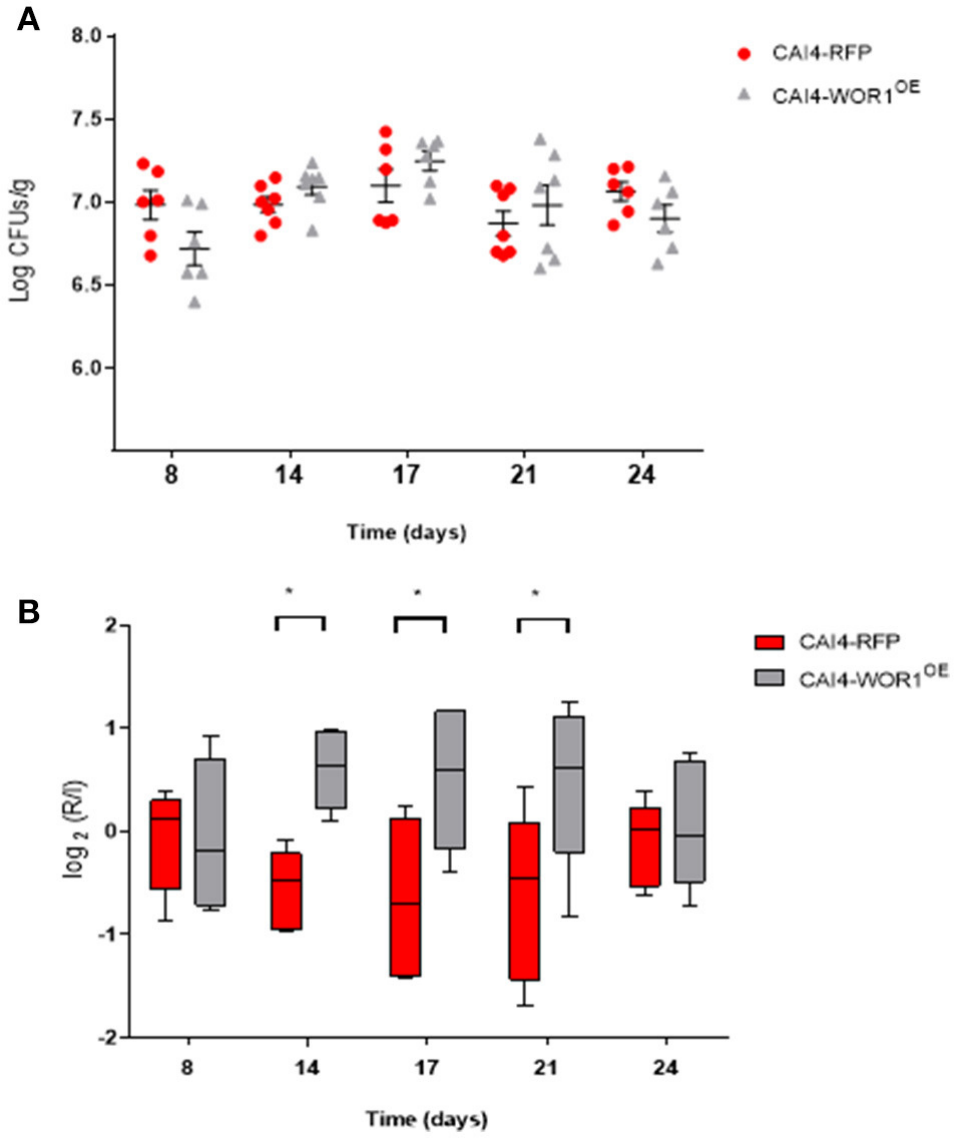
*In vivo* fitness of CAI4-*WOR1*^*OE*^ cells in C57BL/6 mice. Oral antibiotic therapy was given to mice (*n* = 7) starting 4 days before inoculation by gavage of 10^7^ cells of a 1:1 mixture of CAI4-RFP and CAI4-*WOR1*^*OE*^ strain (day 0). CFUs for each strain were counted. **(A)** Fungal loads from fecal samples of each animal and mean ± SEM are plotted at different time points after inoculation (in days). Red symbols correspond to CAI4–RFP while gray symbols to CAI4-*WOR1*^*OE*^ strain. **(B)** The log_2_ of the ratio of cells from fecal samples (R) Vs. the inoculated amount (I) for each strain is represented (box and whiskers, 10–90 percentile) at different time points. ^*^p < 0.05.

In order to determine whether overexpression of *WOR1* generates cells with increased colonization fitness, we performed competitive colonization experiments between CAI4-*WOR1*^*OE*^ and an isogenic strain expressing RFP instead of *WOR1* (Prieto et al., 2014). Analysis of CFUs from stools of mice revealed that colonization of both strains was in the range of 10^7^ CFUs/g; however, strain CAI4-*WOR1*^*OE*^ showed an increased colonization ≈2 weeks after gavage (Figure 1A) that was lost over time. This was more apparent in the relative ratios of both strains determined by the log_2_(R/I) index (Pande et al., 2013) which reflects the ratio of the abundance of each strain compared to the starting (inoculum) amounts (Figure 1B). However, these changes were not maintained at later time points when differences in colonization were statically non-significant after 24 days (*p* = 0.93) and higher times (not shown).

We conclude from these set of experiments that sustained ectopic expression of *WOR1* from the tetracycline promoter results in GUT-like cells *in vitro*, which behave similar to the previously described GUT phenotype. Importantly, we note that increased fitness is not maintained in prolonged colonization studies of greater than 3 weeks.

### *WOR1^*OE*^* results in a short-termed reduced fitness of *C. albicans* cells following oral administration

**Figure 2:**
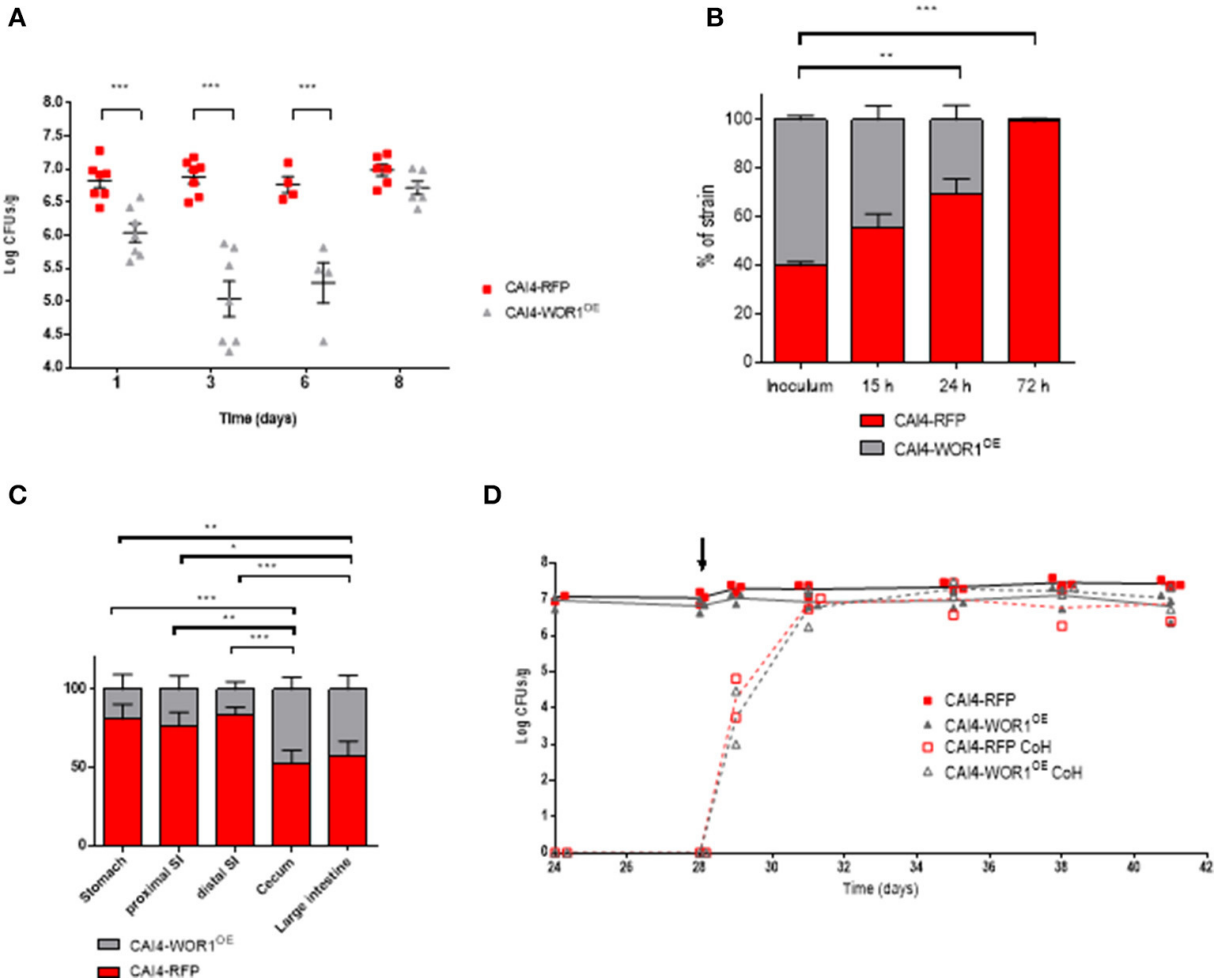
Behavior of CAI4-*WOR1*^*OE*^ cells at early stages of colonization and distribution along the gastrointestinal tract. **(A)** Fungal loads from each individual and mean ± SEM are represented at early stages as red squares (CAI4-RFP) or gray triangles (CAI4-*WOR1*^*OE*^). **(B)** Comparison of percentages of CAI4-RFP and CAI4-*WOR1*^*OE*^ strains in the inoculum and in fecal samples at early time points (mean ± SEM). **(C)** Comparison of percentages of CAI4-RFP and CAI4-*WOR1*^*OE*^ strains in different parts of the gastrointestinal tract. (mean ± SEM). **(D)** Fitness of CAI4-*WOR1*^*OE*^ in co-housing experiments. Oral antibiotic therapy was given to mice (*n* = 3) from 4 days before a gavage of 10^7^ cells in a 1:1 mixture of CAI4-RFP (res squares) and CAI4-*WOR1*^*OE*^ (gray triangles) strains (day 0). On day 28, 2 antibiotic treated mice (open symbols) were placed together with already colonized mice. ^*^p < 0.05, ^**^p < 0.01, ^***^p < 0.001.

While performing competitive colonization experiments we noticed that although precise equal proportions of viable cells (as determined by CFU counting on solid media) of wt and *WOR1*^*OE*^ were adjusted in the inoculum, the proportions of CAI4-*WOR1*^*OE*^ and wt in the first days of colonization were significantly different. This was especially surprising, as fecal CFUs counts during the first days after inoculation closely resemble the initial inoculum dose (Prieto and Pla, 2015). As shown in Figure 2A, colonization initiated with a dose of 10^7^ total cells (1:1 proportion) rendered ≈10^6^ CFUs/g of *WOR1*^*OE*^ at day 1 and ≈10^5^ at day 3, with the ability to reach wt colonization levels observable 2 weeks later. In order to define more precisely this phenomenon, we performed time course experiments for competitive colonization. We analyzed the CFUs of stools at very early times points of 15, 24, and 72 h after gavage (Figure 2B). Although, the inoculum proportions of CAI4-*WOR1*^*OE*^ in the particular experiment shown in the figure was about 60%, it diminished to ≈40% after 15 h, ≈30% after 24 h and to only ≈2% after 3 days. One caution in the interpretation of this data is that CFU actually determines distal (cecum/large intestine) colonization, but the location of the cells (and their relative proportions) could vary between different regions of the intestine regions. We, therefore, performed a *post mortem* analysis of mice colonized simultaneously with both CAI4-*WOR1*^*OE*^ and CAI4-RFP after 28 days, when colonization levels were approximately similar (≈10^7^ CFUs/g). Different regions of the intestine, stomach, proximal small intestine, distal small intestine, cecum, and large intestine, were processed and CFUs were counted. Interestingly, the percentage of *WOR1*^*OE*^ cells varied between 19% in the stomach, 23% in the proximal intestine and 16% in the distal intestine. By contrast we obtained levels as high as 47% in the cecum and 42% in the large intestine (Figure 2C). Therefore, we conclude that a prevalence of wt over CAI4-*WOR1*^*OE*^ occurs in the upper intestinal tract. We finally performed co-housing experiments, where new non-colonized mice are added to the same cage of mice already colonized so that they are inoculated “naturally” via coprophagy. Mice inoculated with a mixture of wt and *WOR1*^*OE*^ cells were co-caged with new mice at day 28. As shown in Figure 2D, CAI4-*WOR1*^*OE*^ and wt cells colonized these new mice with similar efficiency and there was no apparent decrease in CFUs in stools of CAI4-*WOR1*^*OE*^ (see days 29 and 31). Collectively, these experiments indicate that overexpression of *WOR1* results in a “barrier” effect upon entry of *in vitro* cultured cells in the mouse gastrointestinal tract, which is reflected in its preferential colonization of specific gastrointestinal regions.

### *WOR1^*OE*^* cells are sensitive to bile salts

**Figure 3:**
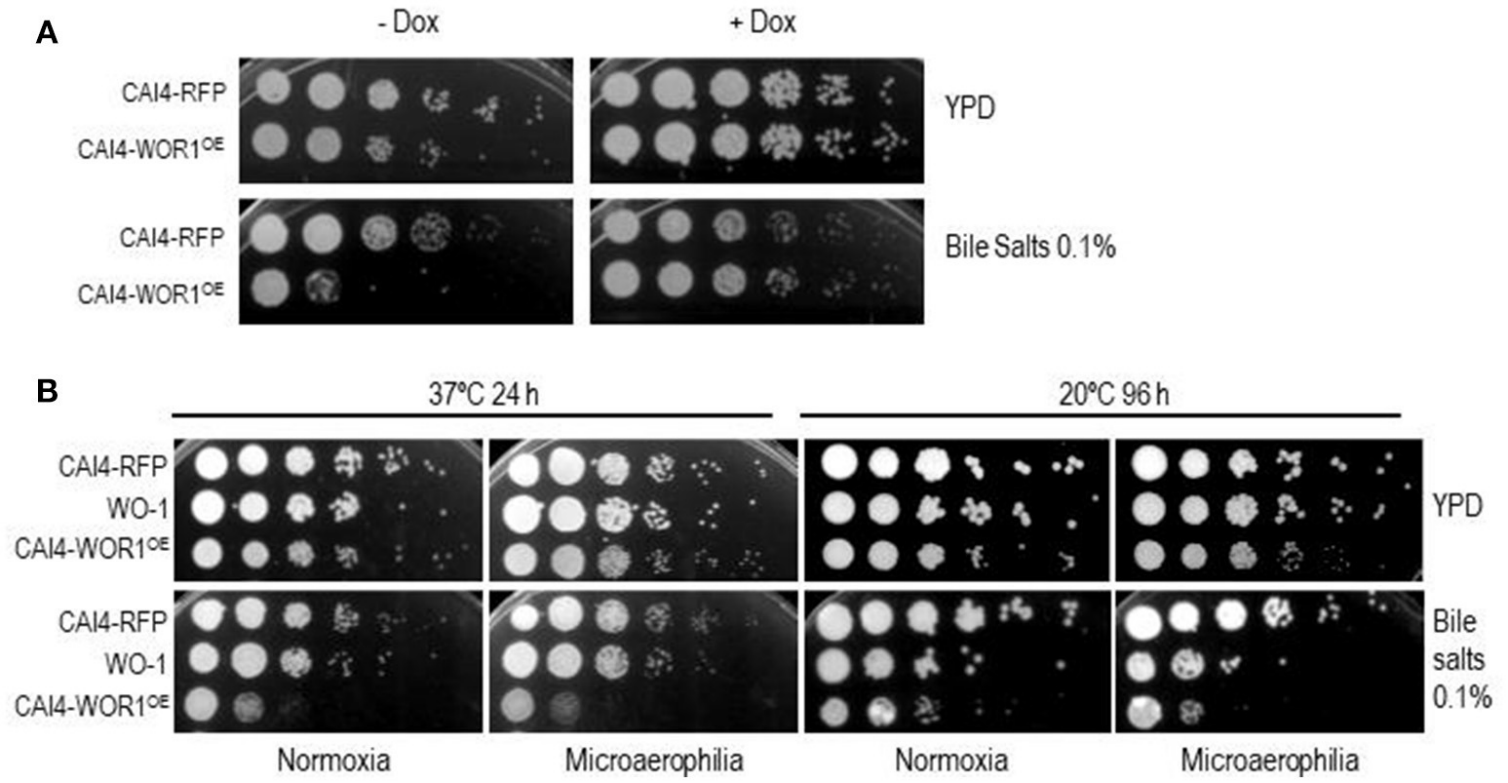
*WOR1* overexpression increases susceptibility to bile salts. **(A)** 10^5^ cells and tenfold dilutions from overnight growing cells in the presence or absence of doxycycline from the indicated strains were spotted onto YPD plates supplemented or not with 0.10% bile salts and in the presence or absence of doxycycline (±Dox). **(B)** Cells from cultures of the CAI4-RFP, CAI4-*WOR1*^*OE*^, and WO-1 strains growing either at 20 or 37°C for 48 h were analyzed as in **(A)**. Plates were incubated at 20 and 37°C and scanned at 96 and 24 h. respectively.

One of the reasons for the altered proportions of *WOR1*^*OE*^ cells in early stools and gut locations could be the susceptibility to bile salts, which are mainly present in the small intestine. We tested this assumption by plating dilutions of overnight growing (YPD) wild type and *WOR1*^*OE*^ cells in solid YPD medium supplemented with bile salts. As observed in Figure 3A, overexpression of both RFP and *WOR1* caused a slight reduction in growth (compare lanes ± DOX), slightly more pronounced in *WOR1*^*OE*^ cells. CAI4-*WOR1*^*OE*^ cells were found to be significantly sensitive to bile salts. This susceptibility is indeed determined by *WOR1* overexpression, as it is dependent on the presence of doxycycline in the medium, which mediates the repression of the ectopic *WOR1* expression and restores wild type bile salt susceptibility (Figure 3A). In order to better mimic the gut atmosphere, we confirmed the phenotype also under oxygen-limiting microaerophilic conditions at 37°C, and noted that the sensitivity seems to be slightly higher than during normoxic growth (Figure 3B). As this phenotype is a new trait assigned to GUT-like cells and given the similarity between GUT and opaque cells (Table 1), we checked whether WO-1-derived opaque cells also shared it. We tested this at 21°C as 37°C forces a conversion of WO-1 opaque cells to white cells. As shown in Figure 3B, WO-1 opaque cells, but not white cells, present a clear sensitivity to bile salts and again, this effect was somewhat increased at low oxygen levels. Therefore, overexpression of *WOR1* leads to susceptibility of cells to bile salts, especially under specific oxygen limiting environments.

### *WOR1^*OE*^* cells have an altered sensitivity to respiratory chain inhibitors

**Figure 4:**
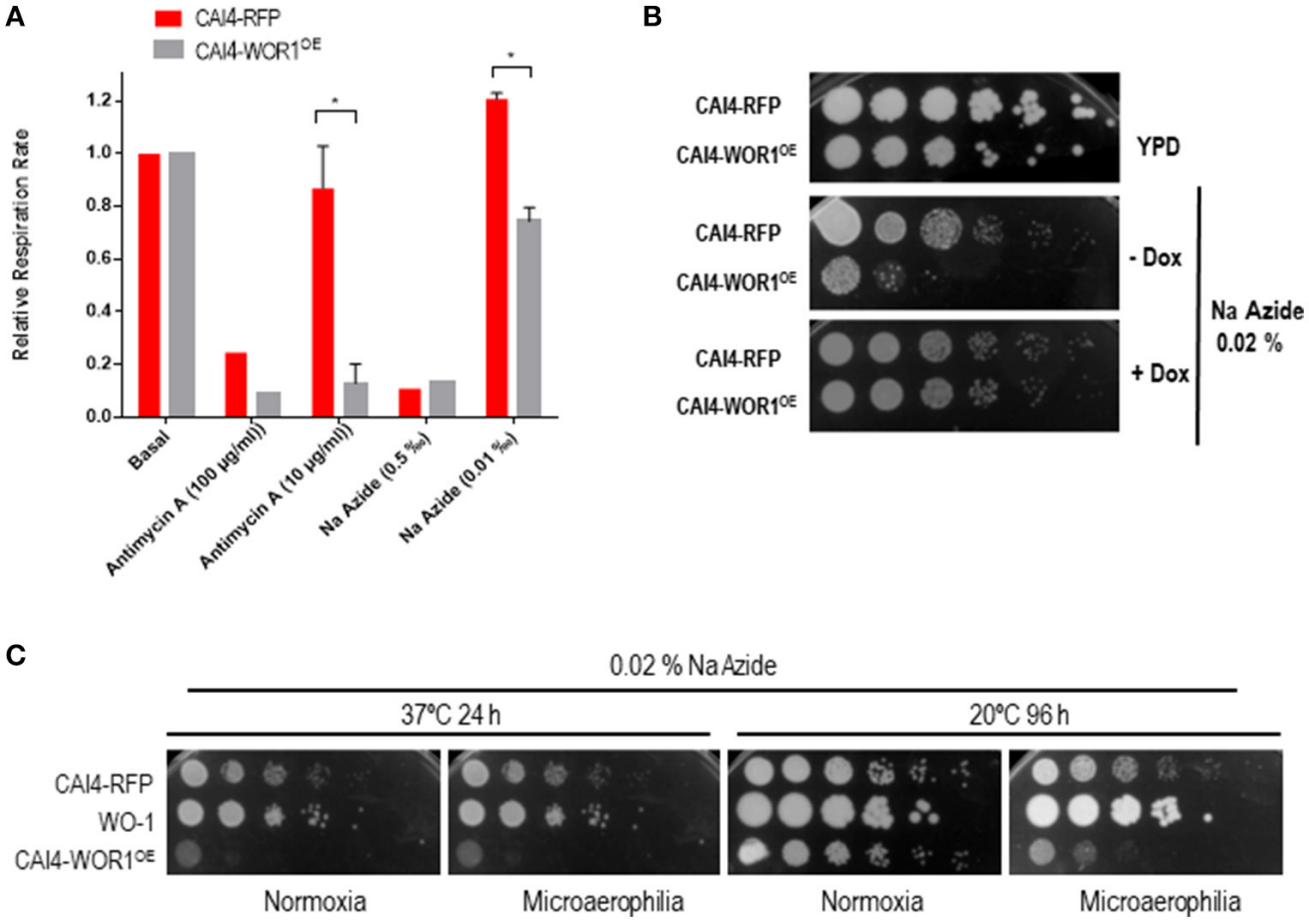
Role of *WOR1* overexpression in oxidative metabolism. **(A)** Inhibition of the basal respiration in the presence of inhibitors of the electron transport chain. O_2_ consumption was quantified by an oxygen electrode and expressed as the ratio of respiration in the presence of compounds vs. basal respiration rate for each analyzed strain. ^*^p < 0.05. **(B)** 10^5^ cells and ten-fold dilutions from overnight growing cells in the presence or absence of doxycycline from the indicated strains were spotted onto plates supplemented with 0.02 ‰ sodium azide (Na Azide) and in the presence or absence of doxycycline (±Dox). **(C)** Cells from cultures of the CAI4-RFP, CAI4-*WOR1*^*OE*^ and WO-1 strains growing either at 20 or 37°C for 48 h were analyzed as in **(B)**. Plates were incubated at 20 and 37°C and scanned at 96 and 24 h respectively in either normoxia or microaerophilia.

A feature of the gut is the limited oxygen availability, especially in the distal portions (He et al., 1999). We reasoned that one of the mechanisms by which GUT-like cells could be adapted to this niche would be a different respiratory metabolism. We, therefore, tested oxygen consumption in wt and *WOR1*^*OE*^ cells in the presence of different inhibitors of respiration. The oxygen consumption of both strains was found to be similar (0.56 ± 0.05 min^−1^/10^6^ cells for wt and 0.59 ± 0.02 min^−1^/10^6^ cells for *WOR1*^*OE*^) under standard laboratory conditions for cells grown in YPG medium (Supplementary Figure 2B). We used Antimycin A and sodium azide to inhibit Complex III and IV of the electron transport chain, respectively (Supplementary Figure 2). When high doses were used, both compounds blocked oxygen consumption of both wt and *WOR1*^*OE*^ strains. Nevertheless, under limiting concentrations of these compounds, the CAI4-*WOR1*^*OE*^ strain displayed a higher sensitivity to both Antimycin A and sodium azide. In the presence of Antimycin A at 10 μg/mL the relative respiration rate values were 0.86 ± 0.16 for CAI4-RFP and 0.13 ± 0.074 % for CAI4-*WOR1*^*OE*^ cells. Cells exposed to 0.01‰ sodium azide displayed a relative respiration rate of 1.2 ± 0.02 in wt cells and 0.74 ± 0.05 in *WOR1*^*OE*^ cells (Figure 4A). In fact, when we tested sodium azide on solid YPD medium, we observed that CAI4-*WOR1*^*OE*^ cells were hypersensitive to this compound, a phenotype that did not occur in the presence of doxycycline (Figure 4B). Interestingly, this feature was not shared by the WO-1 strain (Figure 4C) that seems to be intrinsically more resistant to this drug. Therefore, the respiratory metabolism of CAI4-*WOR1*^*OE*^ is altered compared to wild type cells.

### *WOR1^*OE*^* cells show increased adhesion specifically to intestine

**Figure 5:**
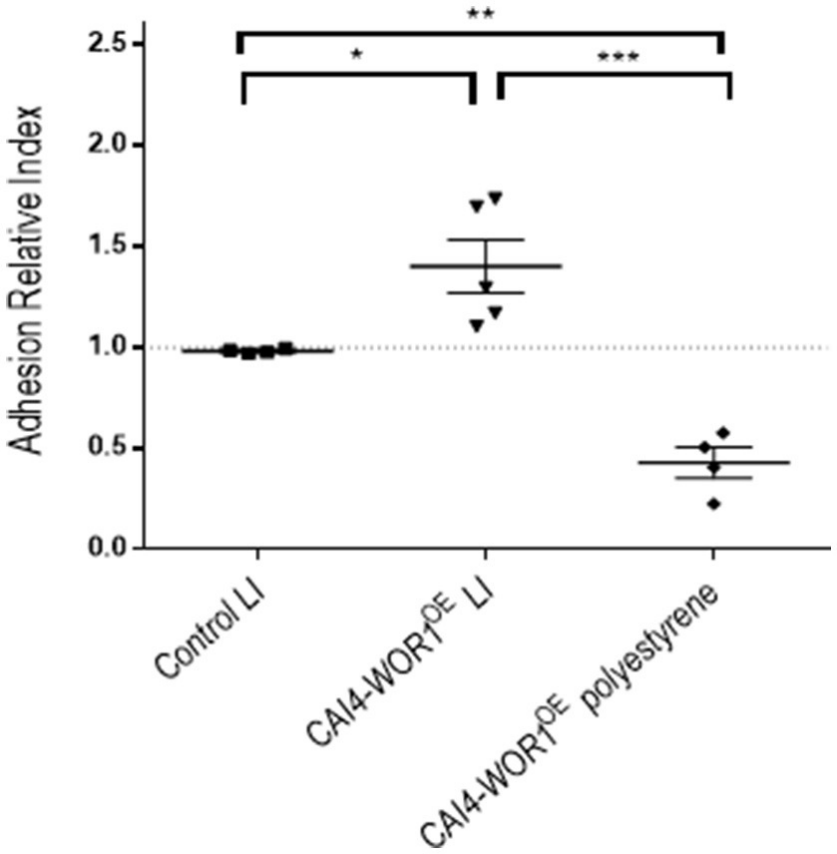
Adhesion of the CAI4-*WOR1*^*OE*^ strain. The Adhesion Relative Index (ARI) was determined for CAI4-*WOR1*^*OE*^ strain in the large intestine (LI) and polystyrene. Individual values are shown with the mean±SEM. ^*^p < 0.05, ^**^p < 0.01, ^***^p < 0.001.

Susceptibility to bile salts does not explain why within a certain range of time (2-3 weeks in our model) colonization of CAI4-*WOR1*^*OE*^ is favored over wt cells. To address this question, we tested the adhesion capacity using a competition assay. In this type of experiments, RFP-labeled wild type cells were used as internal control and mixed in equal proportions with a tester strain. After interaction with the adhesion surface, cells are recovered and the relative proportions of both cells types relative to the inoculum is determined to calculate an Adhesion Relative Index (ARI). This approach results in less experimental variability than that observed in absolute adhesion tests (Prieto et al., 2014). We tested CAI4-*WOR1*^*OE*^ using a biotic (the large intestine mucosa) and an abiotic (polystyrene) surface. *WOR1*^*OE*^ cells showed increased adhesion to the mouse gut mucosa relative to wt cells in the samples that employed large intestine samples with an ARI = 1.40 ± 0.13 (mean ± SEM) (Figure 5). This effect was also observed in the small intestine tissue samples (data not shown). Interestingly, CAI4-*WOR1*^*OE*^ cells showed the opposite effect when tested on 24-well plates (polystyrene abiotic surface), ARI = 0.43 ± 0.08 (mean ± SEM). This indicates that *WOR1* overexpression results in cells with enhanced competitive adhesion to the mouse intestine although this effect is not general to all surfaces.

## Discussion

Colonization of the human gut by *C. albicans* is relevant as many disseminated candidiasis have an endogenous origin (Nucci and Anaissie, 2001; Magill et al., 2006; Miranda et al., 2009). Understanding processes by which this fungus is able to persist within the bacterial microbiome may lead to effective antifungal strategies. The GUT transition was discovered analyzing *C. albicans* mutants with altered fitness in a commensal gastrointestinal mouse model and lead to the view that a continuous expression of WOR1 increased overall fitness (Pande et al., 2013). GUT cells display features different from white and/or opaque cells (Noble et al., 2016). In this work, we generated GUT-like cells by overproduction of the *Wor1* (White Opaque Regulator 1) from the strong doxycycline promoter (Park and Morschhauser, 2005). This GUT-like cells generated *in vitro* are indistinguishable from the already described GUT cells; they keep **a**/α *MTL locus* and display ellipsoid cell shape without pimples on their surface (Table 1). However, these cells demonstrate a decrease in viability upon oral administration resulting in an important unbalance in fitness competition experiments with isogenic parental strains during the first days following colonization. This effect had been described by day 5 (Pande et al., 2013), but is not as drastic as we report here for the very early time points (1–3 days) of colonization we analyzed. This time course is in accordance with the different stages reported for *C. albicans* colonization (Prieto and Pla, 2015). We think that the use of qPCR to quantify the relative amounts of cells may obviously lead to discrepancies due to the presence of dead cells, as these cells have DNA but do not count as viable (CFU+) cells. We propose here that this reduction in colonization could be caused by the intrinsic sensitivity to bile salts of GUT-like cells, a hypothesis that is consistent with the relative proportions we observe along the gastrointestinal tract. Bile salts are an important mechanism of defense against occasional non-intestinal bacteria and have been shown to either aggravate or reduce the symptoms of some intestinal diseases (Hofmann, 1999). This effect was, therefore, unexpected for a typical commensal microbe, as *C. albicans* does not have a significant saprophytic life style outside the human body (Odds, 1988; Noble et al., 2016). It may be relevant in others models of experimental infection. For example, during an experimental disseminated candidiasis in mice, *C. albicans* can be lodged in the gall bladder from where it can be secreted for incorporation into stools (Jacobsen et al., 2014). Lodging in the liver is a risk in liver transplants recipients (Romero and Razonable, 2011) and antifungal treatment in this niche may impose additional problems (Hsieh et al., 2017). Therefore, while more adapted to the gut, GUT-like cells may have reduced ability to colonize specific organs, which is consistent with their reduced competitiveness with the mouse systemic virulence model (Pande et al., 2013). Although we do not know currently the reasons for this sensitivity, unconjugated bile salts (cholate and deoxycholate) interact with lipid membranes in a process which is dependent both on the composition of the membrane and the chemical structure of the bile salt (Begley et al., 2005; Merritt and Donaldson, 2009). An inspection of the *C. albicans* genome does not support the existence of bile salt hydrolases which are, however, present in bacteria such as *Listeria monocytogenes* (Gahan and Hill, 2014). This could suggest changes in membrane composition of GUT-like cells, which is consistent with the already reported transcriptomal analysis of GUT where several fatty acid metabolism genes are altered (Pande et al., 2013). Our co-housing experiments are consistent with the development in GUT cells of fitness traits during growth in the animal (Pande et al., 2013), as these cells do not show an initial reduction in viability.

Our studies also indicate that GUT-like cells have an altered respiratory metabolism *in vitro*, which is reflected by their enhanced sensitivity to inhibitors such as azide. In fact, very low amounts of azide stimulate oxygen consumption in wt cells, as probably expected for a rescue mechanism, but do the opposite in *WOR1*^*OE*^ cells. One explanation for this phenotype could involve the presence of a diminished alternative oxidase pathway (Huh and Kang, 2001) in *WOR1*^*OE*^ cells, rendering cells more dependent on energy production associated with the classical respiratory pathway. While we have been unable to detect such differences *in vitro* regarding SHAM sensitivity (not shown), the atmosphere within the gut (mainly anaerobic or microaerophilic) and the carbon sources are significantly different from those used to culture cells. Another possibility is that adaptation to gastrointestinal tract leads to the selection of cells with low efficient oxidative metabolism, thus sensitive to low amounts of inhibitors. GUT cells differ from opaque and white cells in the expression of glucose catabolism genes (Pande et al., 2013). In a recent study, metabolic differences were detected using an extensive phenotypic profiling between opaque and white cells *in vitro* (Ene et al., 2016) and seem to affect the interaction with the mammalian gut, where nitrogen and carbon sources are different.

Given the partially overlapping common elements that regulate the white/opaque transition, there is a great interest in determining and differentiating their triggering stimuli. The presence of high levels of CO_2_ (equivalent to those found in the host gastrointestinal tract and some tissues), N-acetylglucosamine (a monosaccharide produced primarily by gastrointestinal tract bacteria) and anaerobic conditions, favor the white-to-opaque transition even at 37°C (Ramirez-Zavala et al., 2008; Huang et al., 2009, 2010) suggesting that opaque cells could be optimized for growth in the gut. However, while opaque cells colonize skin more readily, causing cavities in the epithelial layer (Kvaal et al., 1999) they are less virulent than white cells in a mouse model of systemic infection (Kvaal et al., 1997). Opaque are more resistant to phagocyte-mediated killing (Kolotila and Diamond, 1990; Geiger et al., 2004; Lohse and Johnson, 2008; Sasse et al., 2013) revealing differences between white and opaque cell types in the interaction with the host immune system. Interestingly, opaque cells are severely attenuated for commensalism (Pande et al., 2013). In a recent study, natural a/α isolates were found to switch to the opaque phase under conditions that mimic the host environment (CO_2_ and N-acetylglucosamine); white and opaque cells showed a different behavior in fungal burden after systemic infection (Xie et al., 2013) although gut colonization was not tested in this study. Clearly, further experimental work must be done to define the signals that trigger the GUT transition in the gastrointestinal tract and the genes involved in such process in addition to *WOR1*.

In any case, the overexpression of *WOR1* may be advantageous for *C. albicans* to adapt to the murine gut in this model since *WOR1*^*OE*^ cells recover from the initial drop in colonization levels and display an enhanced adhesion to the mouse gastrointestinal mucosa when competing with wild type cells. It has been shown that overexpression of this gene in *Saccharomyces cerevisiae* promotes adhesion to polystyrene (Li and Palecek, 2005) as well as invasion on solid surfaces by overriding of the normally Flo8-dependent Flo11 expression (Huang et al., 2006). In our experiments, the situation is different as *WOR1* diminishes adhesion to polystyrene, at least in competition experiments. It is difficult to draw conclusions from the reported transcriptomal analysis of GUT cells because data were obtained using *in vitro* cultured cells (and not *in vivo*). In addition, adhesion is multifactorial and involves several adhesins with different ligand binding affinities (Chaffin, 2008; de Groot et al., 2013; Hoyer and Cota, 2016) whose relevance in adhesion to mouse mucosa has not experimentally determined. Nevertheless, our results indicate that Wor1-mediated effects are specific and discriminate between an abiotic surface and mucosal tissues. Although differences are not drastic, subtle differences may be relevant during long-term colonization, promoting the presence of a cell reservoir which could involve biofilm formation that would not be efficiently removed by the normal shedding. We do not know the receptors which are specifically targeted by *WOR1*^*OE*^ *in vivo* but one possibility is the mucus layer, counterbalancing the reported reduction in adhesion of *C. albicans* by mucins (Kavanaugh et al., 2014).

Are the traits reported here relevant for the colonization of the gut tract? The current hypothesis about the generation of GUT cells is that within a population, a small percentage of the **a**/α cells have increased levels of Wor1 which in turn (via its positive feedback) triggers the epigenetic conversion to GUT cells. In such scenario, this could occur *in vivo* after a certain period of adaptation, therefore minimizing the role of bile salt sensitivity. However, it could be promoted by multiple signals present there such as glucosamine, CO_2_ and nutrient availability, all which have been described to influence *WOR1* expression, which in turn would allow a more efficient adhesion and metabolic adaptation. The availability of regulated strains where *WOR1* expression can be triggered *in vivo* via doxycycline may facilitate the dissection of *C. albicans* mechanisms of adaptation to the commensal state in a near future.

## Materials and methods

### Strains and growth conditions

The strains used are described in Table 2. Cells were grown at 37°C in YPD medium (1% yeast extract, 2% peptone, and 2% dextrose) unless otherwise stated. Two independent *WOR1*^*OE*^ clones (c1 and c2) were generated which gave similar expression levels and *in vitro* phenotypes (not shown) and only one (c1) was used for *in vivo* studies. The susceptibility/resistance to different compounds was performed through drop test as follows. Cultures grown at 37°C from either stationary or exponential phase (O.D. = 1) were adjusted to 2 × 10^7^ cells/mL, serially 10-fold diluted and deposited (5 μL) onto solid YPD plates supplemented (or not) with the indicated compounds. Plates were incubated at 37°C for 24 and 48 h before scanned. Microaerophilia was achieved using an anaerobic chamber and a commercial system that ensures the adequate percentages of O_2_ and CO_2_ (GENBox Microaer, BioMérieux, ≈15% CO_2_ and ≈6% O_2_)_._ For the observation of white-opaque switching, *C. albicans* strains were grown on YPD plates supplemented with phloxine B (10 μg/mL) at 37 and 21°C. When necessary, doxycycline was added to either liquid or solid media at 10 or 20 μg/mL respectively.

**Table 2:** *Candida albicans* strains used in this work.

**Strain**	**Genotype**	**References**
CAI4	*ura3*Δ*::imm434/ura3*Δ*::imm434*	Fonzi and Irwin, 1993
WO-1	Clinical isolate	Slutsky et al., 1987
CAI4-RFP	[CAI4] *ADH1/adh1::TDH3^*PR*^tTA TET^*PR*^-dTOM2-URA3*	This study
CAI4-*WOR1^*OE*^*	[CAI4] *ADH1/adh1::TDH3^*PR*^tTA TET^*PR*^-WOR1-myc-URA3*	This study

### Genetic procedures

To achieve ORF ectopic expression, RFP labeling and myc fusion, plasmids pNRUX-RFP, and pNRUX-WOR1 were constructed. These plasmids contain a tetracycline repressible promoter. Strains were obtained by integrating ectopically a *Kpn* I-*Sac* II fragment of plasmids pNRUX-RFP or pNRUX-WOR1 in the *ADH1* locus of CAI4. pNRUX-RFP plasmid was generated by replacing the 5′ *ADH1 Xba* I-*Sac* II fragment (880 bp) from pNRU-RFP (Correia et al., 2016) with a 1,630 bp *Xba* I-*Sac* II fragment from the pNIMX vector (Chauvel et al., 2012) containing the 5′*ADH1* and the *TDH3* promoter (*TDH3*^*PR*^). The *WOR1* ORF was amplified by PCR from the clinical isolate SC5314 strain using the primers up-WOR1-myc (GAGATGTCGACAATGTCTAATTCAAGTATAGTCCCTACATATAATG) and rev-WOR1-myc (TCGCGGCCGCGAGTACCGGTGTAATACGACCCAG). The 2364 bp PCR product was cloned in the intermediate pGEMT plasmid (Promega), digested with *Sal* I and *Not* I and accommodated in the pNRUX-RFP, previously digested with *Sal* I and *Not* I to generate the pNRUX-WOR1 vector. Homologous recombination occurs at the *ADH1* locus following a *Kpn* I-*Sac* II digestion and transformation of *C. albicans* using described procedures (Kohler et al., 1997). The correct integration was checked by Southern Blot. *MTL* status was determined by PCR of genomic DNA using the following primers: MTLa-up (TTGAAGCGTGAGAGGCTAGGAG), MTLa-lo (ATCAATTCCCTTTCTCTTCGATTAGG), MTLα-up (TTCGAGTACATTCTGGTCGCG) and MTLα-lo (TTCGAGTACATTCTGGTCGCG).

### Protein extracts and immunoblot analysis

All procedures involving cell lysis, protein extraction, gel electrophoresis, and transfer to nitrocellulose membranes were made as previously described (Martín et al., 1993; Martin et al., 2000). Protein extracts were measured at A_280nm_ to equalize the amount of protein loaded for western blot analysis and Blots were probed with anti-myc, clone 4A6 (Millipore). Western blots were developed according to the manufacturer's conditions using the Hybond ECL kit (Amersham Pharmacia Biotech).

### Measurement of oxygen consumption

*C. albicans* strains were grown in YPG at 30°C until they reached an O.D. ≈1–1.5. Cells from 20–30 mL cultures were recovered by low speed centrifugation (5,000 rpm, 3 min), washed twice with PBS and suspended in 1 mL PBS. To quantify oxygen consumption, an oxygen electrode (Hansatech Instruments) was used. The oxygen electrode chamber was maintained at 30°C, filled with 1 mL PBS and 10 μL of the cell suspension was introduced in the electron chamber. Different compounds that inhibit specific complexes of the electron transport chain were added to the electrode chamber to quantify the contribution of different complexes to the respiration rate. Antimycin A (Sigma-Aldrich) and sodium azide (Panreac) were added at the concentration indicated in the figures to the electron chamber. The relative respiration rate was determined by dividing the respiration rate in the presence of the inhibitor by the respiration rate in its absence (basal).

### *In vivo* fitness assays

The gut colonization assay was performed following the protocol described previously (Prieto et al., 2014). Briefly, after 4 days of antibiotic pre-treatment (2 mg/mL streptomycin, 1 mg/mL bacitracin, and 0.1 mg/mL gentamycin), 10^7^ *C. albicans* cells were intragastrically inoculated by gavage. Stool samples were obtained at different days and homogenized in PBS prior to plating on SD plates with chloramphenicol (20 μg/mL) and YPD plates with phloxine B (10 μg/mL) to quantify CFUs. To analyze *C. albicans* loads in the gastrointestinal tract, mice were sacrificed and samples from the stomach, cecum, small and large intestine were aseptically obtained, homogenized in sterile PBS and cultured in SD plates. Female mice C57BL/6 were purchased from Harlan Laboratories, Inc. (Italy) and used within an age of 7–10 weeks-old. Mice housing and other non-invasive procedures took place in the animal facility at the Medical School of the Universidad Complutense de Madrid. All experiments involving animals performed in this work were carried out in strict accordance with the regulations in the “Real Decreto 1201/2005, BOE 252” for the Care and Use of Laboratory Animals of the “Ministerio de la Presidencia,” Spain. The protocol used in the commensalism model was approved by the Animal Experimentation Committee of the University Complutense of Madrid (CEA 33-2015) and Comunidad de Madrid according to Artículo 34 del RD 53/2013 (PROEX 226/15). The treatments here did not result in disease in the animals; nevertheless, all procedures were conducted minimizing any suffering. The number of animals per experiment was adjusted to a minimum for ethical reasons. Experiments were done at least twice (normally three) and only one representative experiment is shown in the figures.

### Scanning electronic microscopy

CAI4-*WOR1*^*OE*^ cells were incubated in YPD medium for 24 h at 37°C. After washing, cells were fixed overnight at 4°C with 2.5% glutaraldehyde in 0.1 M Na-cacodylate (pH 7.4). Then cells were washed and post-fixed for 2 h in 1% osmium tetroxide in 0.1 M sodium cacodylate buffer (pH 7.4), gradually dehydrated in ethanol and dried. A JEOL JSM-6400 microscope was used for visualization.

### Adhesion assays

Adhesion to polystyrene was performed in 24-well flat bottom plates for culture cells. *C. albicans* strains from overnight YPD cultures were mixed (1:1) and adjusted to 2 × 10^4^ cells/mL concentration. 10^5^ cells were added to each well in YPD medium and allowed to adhere for 90 min at 37°C. Once non-adhered cells were washed out with sterile PBS (3 times), then adhered cells were mechanically removed and spread on SD plates with chloramphenicol (20 μg/mL) for CFUs count. Adhesion to intestinal mucosa we assessed as previously described (Prieto et al., 2014). Briefly, a 1 cm-piece of the large intestine, was opened, washed and placed in a 4 mm-diameter methacrylate chamber, which was filled with RPMI medium pre-warmed at 37°C. Then, *C. albicans* strains from overnight YPD cultures were mixed (1:1) and adjusted to 2.5 × 10^7^ cells/mL concentration in serum-free RPMI medium. 10^6^ yeast cells from this suspension were placed in the lumen side from the colonic tissue and incubated for 150 min at 37°C. Then, the piece of intestine was carefully washed with sterile PBS twice and mechanically disaggregated. This fraction was spread on SD plates with chloramphenicol (20 μg/mL) and YPD plates with phloxine B (10 μg/mL) for CFUs determination. An internal control (CAI4-RFP) was introduced in all adhesion assays; therefore, adherence is quantified by the ARI. This index is calculated by dividing the relative amounts of the strain under analysis in the adhered cells' fraction relative to the value of that same strain in the inoculum.

### Statistical analysis

Statistical differences between two groups were calculated using Student's two-tailed unpaired *t*-tests. Statistical differences between more than two groups were calculated using One-way ANOVA correcting for multiple comparisons using Tukey method. Only *p* < 0.05 were considered significant.

## Author contributions

DP, ER, RM: Experimental work and design, and written, JP: Experimental design, supervisor, and written.

### Conflict of interest statement

The authors declare that the research was conducted in the absence of any commercial or financial relationships that could be construed as a potential conflict of interest.
