# Supplementary figures and images for: Overexpression of the Transcriptional Regulator WOR1 Increases Susceptibility to Bile Salts and Adhesion to the Mouse Gut Mucosa in Candida albicans
Source: Front Cell Infect Microbiol. 2017 Sep 12;7:389. doi: 10.3389/fcimb.2017.00389 (PMC5600957; doi:10.3389/fcimb.2017.00389)

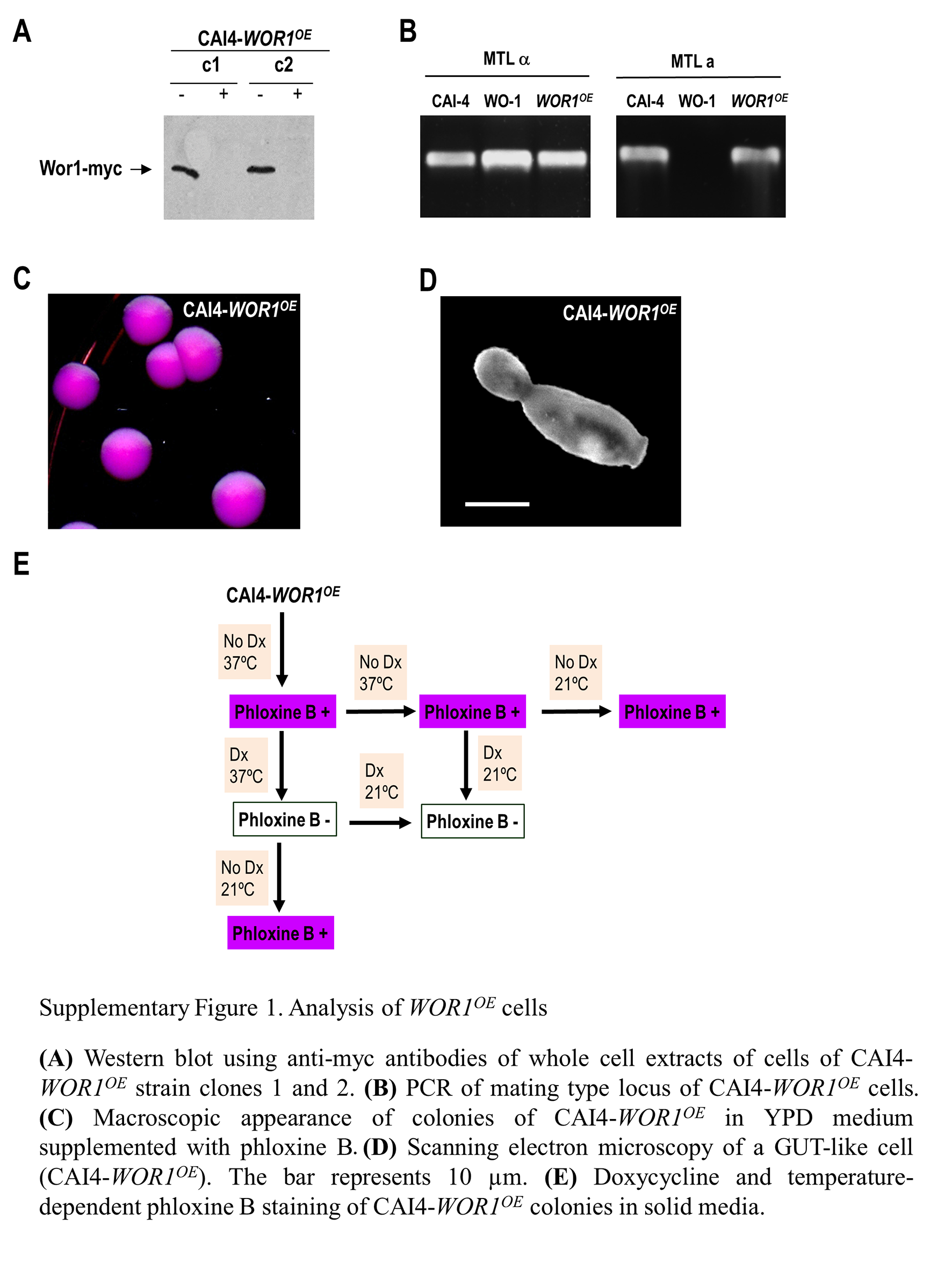

Supplement: Supplementary file 1 [file Image1.TIF]

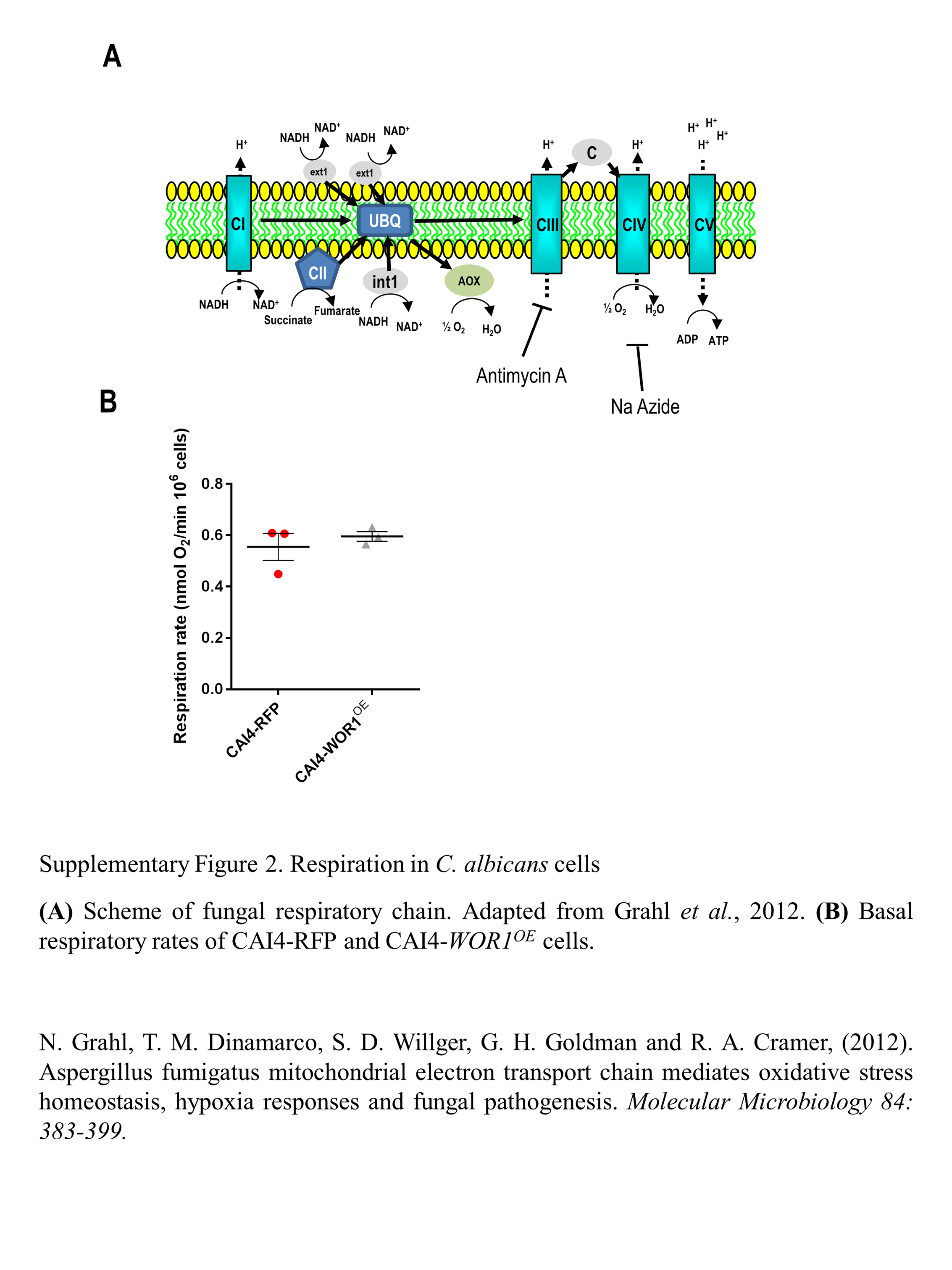

Supplement: Supplementary file 2 [file Image2.tif]
